# Metabolic Control of Innate Lymphoid Cell Migration

**DOI:** 10.3389/fimmu.2019.02010

**Published:** 2019-08-22

**Authors:** Tim Willinger

**Affiliations:** Department of Medicine Huddinge, Center for Infectious Medicine, Karolinska Institutet, Stockholm, Sweden

**Keywords:** innate lymphoid cells, migration, metabolism, oxysterol, inflammation, cancer

## Abstract

Innate lymphoid cells (ILCs) are specialized immune cells that rapidly respond to environmental challenges, such as infection and tissue damage. ILCs play an important role in organ homeostasis, tissue repair, and host defense in the mucosal tissues intestine and lung. ILCs are sentinels of healthy tissue function, yet it is poorly understood how ILCs are recruited, strategically positioned, and maintained within tissues. Accordingly, ILC migration is an area that has recently come into focus and it is important to define the signals that control ILC migration to and within tissues. In this context, signals from the local tissue microenvironment are relevant. For example, ILCs in the intestine are exposed to an environment that is rich in dietary, microbial, and endogenous metabolites. It has been shown that the Vitamin A metabolite retinoic acid promotes ILC1 and ILC3 homing to the intestine. In addition, recent studies have discovered cholesterol metabolites (oxysterols) as a novel class of molecules that regulate ILC migration through the receptor GPR183. ILCs are considered to be largely tissue-resident cells, yet recent data indicate that ILCs actively migrate during inflammation. Furthermore, the discovery of circulating ILC precursors in humans and their presence within tissues has fueled the concept of local ILC-poiesis. However, it is unclear how circulating ILCs enter tissue during embryogenesis and inflammation and how they are directed to local tissue niches. In this review, I will discuss the metabolic signals that regulate ILC homing and their strategic positioning in healthy and inflamed tissues. It is becoming increasingly clear that ILC function is closely linked to their tissue localization. Therefore, understanding the tissue signals that control ILC migration could open new avenues for the treatment of chronic inflammatory diseases and cancer.

## Background

Innate lymphoid cells (ILCs) are immune cells of lymphoid origin that quickly respond to perturbations of tissue homeostasis. Apart from their role in barrier immunity and host defense, ILCs are also essential for organ homeostasis, recovery from tissue injury, and metabolism (1–5). In addition to cytotoxic natural killer (NK) cells, three different ILC types can be distinguished based on signature transcription factors and effector cytokines, similar to CD4^+^ T helper lymphocytes: (1) T-BET^+^ ILC1s produce interferon-gamma (IFNγ); (2) GATA3^high^ ILC2s produce interleukin-5 (IL-5) and IL-13; (3) RORγt^+^ ILC3s produce IL-17 and/or IL-22. RORγt^+^ ILC3s include fetal lymphoid tissue-inducer (LTi) cells and adult LTi-like cells that have a similar phenotype (CCR6^+^NKp46^−^) and mainly reside in lymphoid tissues (6, 7). LTi cells are now considered a separate ILC lineage due to their unique ontogeny (5, 8). α4β7^+^CXCR6^+^ ILC3 precursors (ILC3Ps) develop into LTi cells in the fetal liver (9). In contrast, adult LTi-like ILC3s can derive from bone marrow precursors that upregulate RORγt in peripheral tissues, such as the intestine, in a Notch-dependent manner (9). Perinatal RORγt^+^ ILCs give rise to long-lived ILC3s in the small intestine (10), yet it is unclear whether and to what extent embryonic LTi cells persist in the adult. Therefore, the developmental relationship between fetal LTi cells and adult LTi-like ILC3s remains to be defined. Adult mice also have T-BET-expressing CCR6^−^NKp46^+^ ILC3s that are derived from CCR6^−^NKp46^−^ ILC3s (11). Dietary phytochemicals acting through the aryl hydrocarbon receptor (AHR) are required for the post-natal expansion of these CCR6^−^ adult ILC3s (12–14). Both fetal LTi cells (15) and adult ILC3s in the intestine (16, 17) are dependent on the Vitamin A metabolite retinoic acid.

Many features of ILCs are shared with T cells, but ILCs also have unique, non-redundant, functions, such as the ability to orchestrate the formation of lymphoid tissues, which is carried out by ILC3s with LTi function (12, 13, 18–21). In mice, the prenatal formation of lymphoid tissues (lymph nodes and Peyer's Patches) is carried out by CD4^+^ fetal LTi cells, whereas adult ILC3s mediate the development of intestinal cryptopatches and isolated lymphoid follicles that develop after birth (22, 23). Besides their beneficial effects, ILCs have been implicated in chronic inflammatory responses that underlie human disease (24–26).

**Figure 1:**
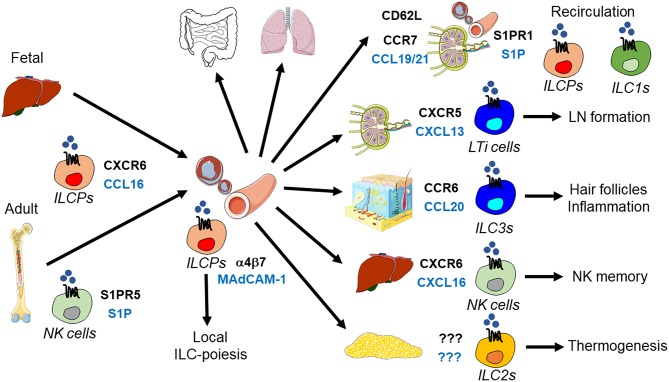
Tissue homing and positioning of ILCPs and mature ILCs. ILCPs derived from fetal (liver) or adult (bone marrow) hematopoiesis enter the blood to home to lymphoid and non-lymphoid organs. This process requires CXCR16 and α4β7 integrin, which bind to CXCL16 and MAdCAM-1, respectively. NK cells egress from the bone marrow is mediated by S1PR5-S1P. Recent data support the concept that circulating ILCPs are a source of local ILC-poiesis after their migration into tissues. ILCPs and ILC1s recirculate between lymph nodes and blood through the use of CD62L, CCR7, and S1P receptors. During embryogenesis, LTi cells are essential for lymph node (LN) formation, which is initiated by their CXCR5-CXCL13-dependent clustering at lymph node anlagen. CCR6-CCL20 positions ILC3s to hair follicles and recruits ILC3s during skin inflammation. In the liver, CXCR6 and its ligand CXCL16 are essential for NK cell memory responses. ILC2s promote thermogenesis, but the signals regulating their migration in adipose tissue are unknown.

**Figure 2:**
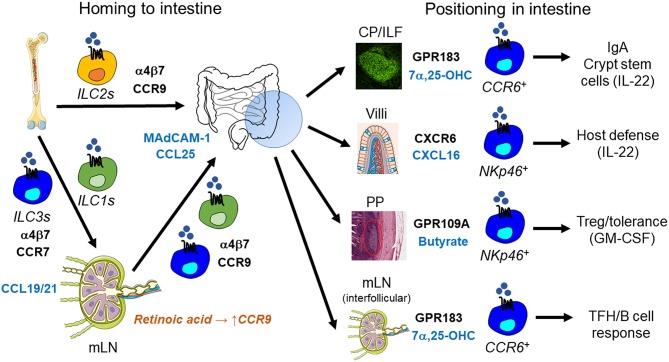
ILC trafficking and positioning in the intestine. ILC homing to the small intestine is mediated by α4β7 integrin and the chemokine receptor CCR9 and their respective ligands MAdCAM-1 and CCL25. ILC2s in the bone marrow already express CCR9 and are therefore capable of homing to the small intestine directly. In contrast, retinoic acid is required to induce CCR9 expression in ILC1s and ILC3s after their CCR7-dependent trafficking to mesenteric lymph nodes (mLN). After homing to the intestine, ILC3s are positioned within different tissue niches by distinct signals: (i) CCR6^+^ LTi-like ILC3s localize to cryptopatches (CP) and isolated lymphoid follicles (ILF) through the GPR183-mediated sensing of 7α,25-dihydroxycholesterol (7α,25-OHC). CP/ILF-resident ILC3s stimulate IgA production by interacting with B cells and support crypt stem cells through the constitutive production of IL-22. (ii) NKp46^+^ ILC3s are positioned to intestinal villi through CXCR6-CXCL16, which promotes host defense against intestinal pathogens via IL-22. (iii) Microbiota-produced butyrate regulates the regional residence of NKp46^+^ ILC3s in Peyer's Patches (PP), which controls intestinal tolerance through GM-CSF secretion and regulatory T cells (Treg). (iv) GPR183 and its ligand 7α,25-OHC are also essential for the localization of CCR6^+^ LTi-like ILC3s to the interfollicular region within mLNs, where they interact with T follicular helper cells (TFH) and B cells to regulate IgA production.

**Figure 3:**
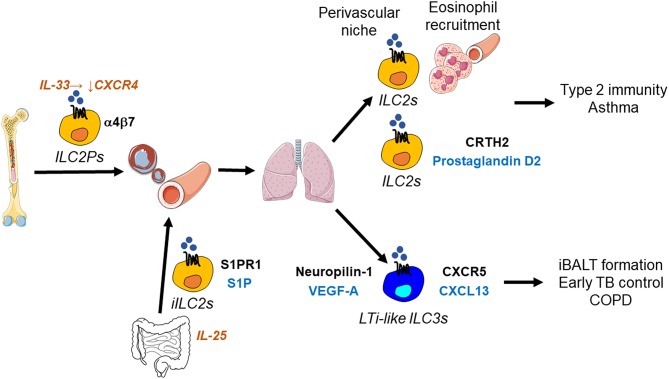
ILC migration in the lung. Suppression of CXCR4 by IL-33 enables ILC2Ps to egress from the bone marrow and home to the lung, where they occupy a perivascular niche. This strategic localization likely allows ILC2-mediated recruitment of eosinophils from the circulation into the lung. In addition, prostaglandin D2 stimulates ILC2 migration in the lung through the interaction with its receptor CRTH2 on ILC2s. ILC2s thereby promote type 2 barrier immunity, but also allergic inflammation as in asthma. During helminth infection, IL-25-responsive inflammatory ILC2s (iILC2s) are capable of trafficking from the intestine to the lung, where they support anti-helminth immunity. This inter-organ trafficking requires S1PR1-S1P. LTi-like ILC3s migrate in the lung using CXCR5-CXCL13 and likely neuropilin-1-VEGF-A. LTi-like ILC3s mediate the formation of inducible bronchus-associated lymphoid tissue (iBALT), which is essential for the early control of tuberculosis (TB) in the lung, but may also be involved in inflammatory responses occurring in chronic obstructive pulmonary disease (COPD).

The main focus in the research on ILCs has been on cell lineage relationships, transcription factors, and effector function—mostly based on analogies with T lymphocytes. ILC migration has only recently become an active area of investigation. Their strategic position within tissues allows ILCs to fulfill their role as sentinels of healthy tissue function. Furthermore, local ILC clustering and rapid migration in response to inflammatory signals may explain why ILCs exert such powerful effects on tissue immunity (Figures 1–**3**). However, much remains to be learned about the pathways that regulate the migration and tissue localization of ILCs. In this review, I mainly discuss the migration of ILCs other than NK cells.

## Tissue Distribution of ILCs

ILCs are found in many organs, but are enriched in mucosal tissues (intestine, lung) that are most exposed to the environment (10, 16, 27–31). Furthermore, the relative abundance of ILC subsets differs between tissues in mice (10, 16, 28) and humans (30, 31), with ILCs perhaps less compartmentalized in humans than in mice (31). For example, ILC3s are abundant in the small intestine and ILC2s in the skin as well as in adipose tissue, whereas NK cells predominate in bone marrow, spleen, liver, and lung (32). In addition, there are regional differences in ILC distribution within the same organ. For example, NKp46^+^ ILC3s are enriched in the small intestine, whereas in the colon adult LTi-like ILC3s are more prevalent. In addition, ILC2s are more abundant in the colon than in the small intestine (32). Moreover, ILC abundance differs between steady-state and inflamed tissue (33–35). Finally, developmental age of the organism influences ILC tissue distribution. For example, LTi-like ILC3s are present in the fetal gut, whereas NKp46^+^ ILC3s are largely absent (10), only expanding after birth in response to diet-derived AHR ligands (12–14) and signals from the maternal microbiota (36). Similarly, ILC2s seed the mouse lungs within the first 2 weeks of life (37). The differential tissue distribution of ILCs is likely related to their migratory behavior, e.g., due to temporal seeding of tissues during embryogenesis (10, 38) and due to organ-specific expression of integrins and chemokine receptors on ILCs (Figures 1–**3**).

Parabiosis studies in mice established the concept that, in contrast to NK cells, ILCs in both lymphoid and non-lymphoid tissues are largely tissue-resident cells (39). This implies that, similar to tissue macrophages, ILCs are maintained within tissues by local self-renewal. However, recent studies have challenged this concept with the discovery of circulating CD117^+^ ILC precursors (ILCPs) in humans (40) and the observation that inflammatory ILC2s in the mouse can migrate from the intestine to the lung during helminth infection (41). Circulating ILCs might therefore constitute mobile a pool of cells that can be activated and recruited to inflamed tissue on demand in order to support host defense carried out by tissue-resident ILCs (Figures 1, **3**). Apart from ILCPs, human blood also contains ILC2s (27), but no mature ILC1s and ILC3s (40). In addition to more abundant NK cells, circulating putative ILCPs and mature ILCs, mainly ILC1s, are also found in mice (42, 43).

Interestingly, ILCs occupying vascular vs. tissue compartments seem to have distinct functions. A recent study demonstrated that NK cells circulating between blood and peripheral tissues have effector function, whereas NK cells trafficking to lymph nodes are long-lived and proliferative (44). This different migratory and functional behavior has been associated with the differential expression of transcription factors (44).

## Tissue Niches and ILC Function

Like other immune cells, ILCs occupy distinct niches within tissue, which is important for their function and likely regulates their homeostasis. For example, in the intestine, ILCs reside in three main anatomical compartments: (i) LTi-like ILC3s are clustered in lymphoid tissues, such as cryptopatches and isolated lymphoid follicles (45, 46); (ii) NK cells/ILC1s, ILC2s, NKp46^+^ ILC3s are dispersed in the lamina propria (47–49); (iii) Intraepithelial ILC1s are located within the epithelium (30, 50). This anatomical compartmentalization corresponds to the diversity of ILC function in the intestine (Figure 2). For example, LTi-like ILC3s in Peyer's Patches and isolated lymphoid follicles interact with B cells to stimulate IgA production (51, 52), which promotes host commensalism with the local microbiota (53). Furthermore, lymphoid tissue-resident commensal bacteria are contained by IL-22-producing ILC3s (54). In addition, ILC3s in cryptopatches are in close proximity to the cypts, where intestinal stem cells reside. Accordingly, IL-22 production by ILC3s has been shown to maintain crypt stem cells after tissue damage (55–57). In contrast, NKp46^+^ ILC3s are mostly resident in the small intestinal villi, located close to the epithelium, where they mediate host defense against pathogens (58). Interestingly, NKp46^+^ ILC3s seem to produce IL-22 mainly in response to pathogen-induced IL-23 secretion by myeloid cells, whereas lymphoid tissue-resident LTi-like ILC3s produce IL-22 constitutively in a microbiota-dependent manner (59). Finally, IFNγ-secreting ILC1s within the intraepithelial compartment are involved in colitis (50).

Although intestinal ILC3s are tissue-resident (39), they are not completely sessile cells. For example, in the steady state, there is a constant influx and egress of ILC3s to and from cryptopatches and there is increased ILC3 mobilization from cryptopatches during inflammation (21, 60). The significance of steady-state ILC3 migration in and out of cryptopatches is unknown, but could potentially serve the purpose of sampling or sensing cues from the environment (such as crypt material) to respond to perturbations of the intestinal stem cell compartment. Accordingly, it has been suggested that cryptopatches act as a platform to rapidly amplify ILC-mediated immune responses, not only through cytokine production, but also through ILC movement into surrounding tissue (60).

Interestingly, occupancy of tissues niches by ILCs is regulated by quorum sensing-like mechanisms. Thus, it has recently been shown that receptor activator of nuclear factor kappa B (RANK)-RANK ligand (RANKL) interactions adjust the numbers of mouse CCR6^+^ LTi-like ILC3s to the size of the niche, likely within cryptopatches (61). Therefore, regardless of their outer environment, clustering of CCR6^+^ ILC3s allows them to keep one another in check. This mechanism likely operates also in human tonsil, where CCR6^+^ ILC3s express both RANK and RANKL (61).

In many tissue niches, ILCs have an intimate relationship with non-hematopoietic cells, such as stromal cells. For example, an ILC3-stromal cell niche in secondary lymphoid organs has been reported in both mice and humans (62). Moreover, ILC2s occupy a distinct perivascular localization close to stromal cells in the lung (63, 64). In this specific niche, adventitial stromal cells promote ILC2 homeostasis in steady-state and in response to helminth infection through the production of IL-33 and thymic stromal lymphopoietin (TSLP) (64). In turn, ILC2-derived IL-13 supports the expansion and IL-33 production by adventitial stromal cells (64). The close proximity of lung ILC2s to blood vessels has been proposed to allow efficient recruitment of eosinophils from the blood (63), further underscoring the importance of ILC intra-tissue localization (Figure 3). Furthermore, ILC2s are strategically positioned within the airways, near airway branch points (65), where inhaled particles are thought to accumulate. This puts ILC2s in close contact with neuroendocrine cells that activate ILC2s through the release of calcitonin gene-related peptide (65). ILC-neuron interactions also occur in intestinal cryptopatches (66) and accordingly neuronal circuits have been shown to regulate ILC function in different contexts (66–71).

Furthermore, there is the emerging concept that different tissue microenvironments specify ILC function, as has been shown for ILC2s (72). Local tissue niches might also regulate ILC function through stimulating ILC plasticity. *In vitro*, ILC plasticity occurs through the exposure to polarizing cytokines, such as IL-1β and IL-12, which induces the conversion of ILC2s and ILC3s into ILC1s (35, 73–76). However, it is unclear where these factors are produced *in vivo* and where ILC trans-differentiation occurs within tissue. One possibility is that migratory signals induced by inflammation guide ILCs to specific niches, where they are exposed to polarizing cytokines. Alternatively, the polarizing cytokines might be produced within the same niche in response to inflammatory stimuli.

Overall, the signals and migratory receptors regulating the co-localization and interaction of ILCs with stromal cells and other immune cells, such as T cells, are largely unknown. Subsets of ILCs interact with T cells through the expression of major histocompatibility complex (MHC) class II, CD1d, OX40 ligand (OX40L), and CD30 ligand (CD30L) (77). In the intestine, MHC class II^+^ ILC3s suppress CD4 T cell responses against the local microbiota (78, 79), whereas the interaction of MHC class II^+^ ILC2s with CD4 T cells promotes type 2 immunity in the lung (80). Moreover, OX40L-expressing ILC2s stimulate Th2 and regulatory T cell (Treg) responses in lung and adipose tissue (81). Interestingly, adult LTi-like ILC3s constitutively express co-stimulatory ligands (OX40L, CD30L), whereas fetal LTi cells do not (82).

Another interesting area for future investigation is the occupation of tissue niches by ILCs and their adaptive counterparts, i.e., T cells. This is particularly relevant since both ILCs and T cells largely dependent on the same factors (γ_c_ cytokines) for their homeostasis and expansion, therefore likely competing with each other. Accordingly, intestinal ILC2s and ILC3s expand in T cell-deficient mice, e.g., in mice lacking *Rag* genes (16), most likely due to increased availability of IL-2 and IL-7. This notion is supported by the finding that intestinal ILC3s outcompete T cells for IL-2 (79) and that IL-7 consumption by ILCs regulates the amount of IL-7 that is available to T cells (83). Finally, it has been suggested that IL-2 produced by proliferating T cells maintains LTi-like ILC3s in lymphoid structures (77), in accordance with the observation that mesenteric lymph node-resident ILC3s are reduced in T cell-deficient mice (84). However, in many tissues it has not been thoroughly investigated whether ILCs and T cells occupy distinct or overlapping niches.

## ILC Trafficking to Tissues

### Trafficking Receptors on ILCs

Mature ILCs are largely tissue-resident cells (39), yet the signals that control the migration of ILCPs and mature ILCs into tissues during embryogenesis, adult life, and inflammation are still incompletely understood. Similar to T lymphocytes, ILC trafficking to tissues is regulated by integrins and chemokine receptors (Table 1) that are often expressed in an ILC subset-specific manner with similar chemokine receptor expression as the corresponding T helper subsets (32).

**Table 1:** Receptors involved in ILC migration.

**Receptor**	**Ligand**	**Source**	**Function**	**References**
**INTEGRINS/SELECTINS**				
α4β7 integrin	MAdCAM-1	Endothelial cells	LTi cell entry into embryonic LNs; ILC trafficking to small intestine and colon	(18, 85)
CD49a	Collagen	Tissue matrix	ILC tissue retention?	(30, 86)
αEβ7 integrin	E-cadherin	Epithelial cells	ILC1 interaction with intestinal epithelium?	(50)
CD69	NA	NA	Inhibition of ILC tissue egress by antagonizing S1P receptors	(41)
CD62L (L-selectin)	MAdCAM-1 GlyCAM-1 CD34	Endothelial cells	LN entry and recirculation of ILCPs, ILC1s, NK cells	(42, 43)
**CHEMOKINE RECEPTORS**				
CCR6	CCL20	Epithelial cells	ILC3 positioning to hair follicles; recruitment into inflamed skin	(43, 87)
CCR7	CCL19/CCL21	Stromal cells	LTi cell clustering at LN anlagen; ILC1/ILC3 migration from BM to mLNs; ILC LN entry and recirculation; ILC3 trafficking from intestine to mLNs; ILC3 recruitment to tumors	(43, 84, 85, 88–90)
CCR9	CCL25	Epithelial cells	ILC trafficking to small intestine	(85)
CXCR4	CXCL12	Reticular cells?	Inhibition of ILC2P egress from BM	(37)
CXCR5	CXCL13	Stromal cells	LTi cell clustering at LN anlagen; ILC3 recruitment to infected lung in tuberculosis; ILC3 clustering with stromal cells in tumors	(88, 90, 91)
CXCR6	CXCL16	CX3CR1^+^ DCs Other cells?	Positioning of NKp46^+^ ILC3s to small intestinal villi; NK cell homing to liver; ILC3P migration from fetal liver to periphery; ILCP egress from BM	(58, 92, 93)
**OTHER RECEPTORS**				
GPR109A	Butyrate	Microbiota	Regional ILC3 distribution in PPs	(94)
GPR183	7α,25-OHC	Stromal cells	ILC3 migration to cryptopatches; ILC3 recruitment to small intestine; ILC3 positioning within mLNs	(21, 95, 96)
S1P receptors	S1P	Red blood cells? Endothelial cells?	NK cell egress from BM and LNs; ILC egress from LNs; ILC2 inter-organ trafficking in helminth infection	(41, 42, 97)
CRTH2	Prostaglandin D2	ILC2s?	ILC2 recruitment to inflamed lung	(98)
Neuropilin-1	VEGF-A	Unknown	LTi cell recruitment to iBALT?	(99)

*BM, bone marrow; iBALT, inducible bronchus-associated tissue; LN, lymph node; mLN, mesenteric lymph node; PP, Peyer's Patch*.

For example, LTi-like ILC3s express CCR6 and CXCR5 (10, 11), both transcriptional targets of RORγt, which are also preferentially expressed on Th17 cells (CCR6) and T follicular helper cells (CXCR5). CCR6 and CXCR5 are already expressed on ILC3Ps that migrate to peripheral tissues from the fetal liver and adult bone marrow (9). In contrast, NKp46^+^ ILC3s express CXCR6 (29, 92), the receptor for CXCL16, which mediates their localization to the lamina propria (58). ILC1s also express CXCR6 (92). Furthermore, CXCR6 promotes the homing of NK cells to the liver, which is important for NK cell memory (93). Lymphoid tissue-resident human ILC3s with LTi activity, as well as murine fetal CD4^+^ LTi cells, not only express CCR6 and CXCR5, but also Neuropilin-1, which mediates their migration toward vascular endothelial growth factor-A (VEGF-A) (99). Finally, distinct subsets of intestinal ILC3s express CD49a (integrin α1) (86). Moreover, similar to Th2 cells, both mouse and human ILC2s express CCR4 (27, 29, 100) and other skin-homing receptors, such as cutaneous leukocyte-associated antigen (CLA) and CCR10 that bind to endothelial cell-leukocyte adhesion molecule 1 (ELAM-1) and CCL27/CCL28, respectively (32). It has been reported that ILC2s in broncho-alveolar lavage fluid highly express CCR4 (and CCR7) after IL-33 administration (101), suggesting a role for CCR4 and its ligands CCL17 and CCL22 in ILC2 migration following activation, although this prediction requires experimental validation. CCR8 is another chemokine receptor that shows shared expression in ILC2s and Th2 cells (29), which may mediate ILC homing to the skin in response to CCL1. In contrast, ILC1s and NK cells share preferential expression of CXCR3, the receptor for CXCL9, CXCL10, and CXCL11, with Th1 cells (102). Furthermore, expression of CD49a and CD49d (integrin α4) can be used to distinguish subpopulations of intestinal ILC1s (86). NK cells also express integrins, such as CD49a and CD49b (integrin α2) (32). In addition to subset-specific expression, migratory receptors are also expressed in a tissue-specific manner within the same ILC subset. For example, ILC2s in adipose tissue have higher expression of *Itgae, Ccr6*, and *Cxcr4* than ILC2s from other tissues (72).

### Seeding of Tissues With ILCs During Development

In mice, ILCs derived from fetal liver hematopoiesis are among the first lymphocytes to seed barrier tissues, such as the intestine before birth (10, 12, 38) (Figure 1). This tissue seeding prepares the host for the colonization of the intestine with the microbiota and the intake of food-derived antigens. Moreover, LTi cells populate organs early to promote the formation of lymphoid tissues (22). ILCPs express α4β7 integrin, whose ligand MAdCAM-1 is widely expressed in the fetus, thereby allowing ILCP migration to a variety of tissues (32). The entry of LTi cells into embryonic lymph nodes is also dependent on α4β7 integrin (103). Furthermore, the interaction of CXCL13, induced by retinoic acid in mesenchymal organizer cells, with CXCR5 on LTi cells is required for the clustering of LTi cells at embryonic lymph node anlagen and lymph node development, with a minor contribution of CCL21 and its receptor CCR7 (88). Further work showed that the amount of maternal retinoic acid regulates the number of LTi cells and therefore the size of lymph nodes, which determines anti-viral immunity later in life (15). Before birth, LTi cells also cluster at embryonic anlagen to promote the formation of Peyer's Patches, which is dependent on expression of RET, a tyrosine kinase receptor for neurotrophic factors, on LTi cells (104). In addition, arginase 1-expressing ILCPs accumulate at Peyer's Patch anlagen, where they can give rise to ILC1s, ILC2s, and ILC3s in the fetal intestine (38). This ILCP clustering occurs in a CXCR5- and CCR7-independent manner, since, in contrast to LTi cells, these Arginase 1^+^ ILCPs do not express CXCR5 and CCR7 (38). In contrast to LTi cells, Arginase 1^+^ ILCPs also lack lymphotoxin expression and are therefore dispensable for Peyer's Patch organogenesis (38). Finally, fetal α4β7^+^CXCR6^+^CCR6^+^CXCR5^+^ ILC3Ps migrate from fetal liver to lymphoid organs and intestine (9, 105) in a CXCR6-dependent manner (92). ILCs are also found in human fetal tissues (27), suggesting that early colonization of tissues with ILCs is conserved between mice and humans.

Overall, embryonic tissue seeding of ILCPs is reminiscent of the colonization of tissues with embryonically-derived macrophages (106, 107). In contrast to organs that are seeded before birth (e.g., the intestine), other organs, such as the lung and spleen, are colonized with ILCs after birth. Later, during adult life, there is likely a second wave of ILCPs from bone marrow (or other tissues) that enter the circulation and gain access to tissues to contribute to the ILC pool found in peripheral organs. Again, this might be in analogy to macrophages, where, in the adult organism, circulating monocytes enter tissues and, under specific conditions, can differentiate into tissue-resident macrophages.

### ILC Trafficking to Lymph Nodes

In adult mice, ILCs use similar mechanisms as naïve T cells for lymph node entry (Figure 1). For example, like NK cells, ILCs (especially ILCPs and ILC1s) enter peripheral lymph nodes using CD62L (L-selectin) and CCR7 (42, 43). In addition, LTi-like ILC3s are capable of trafficking from the intestine to draining mesenteric lymph nodes in a CCR7-dependent manner (84). In contrast, ILC1s, ILC2s, and NKp46^+^ ILC3s do not migrate along this route. Accordingly, LTi-like ILC3s migrate toward the CCR7 ligand CCL21 *in vitro*, whereas ILC2s are unable to do so (84). Finally, the trafficking of LTi-like ILC3s to the spleen is not critically dependent on CCR7 (84).

### ILC Trafficking to the Intestine

Tissue-specific signals from the local microenvironment likely play an important role in the trafficking of ILCs to the intestine, including cues from the microbiota, which might be particularly important for intestinal ILC3s (36, 86). In addition, metabolic cues are essential as has been demonstrated in a few studies so far (Figure 2). For example, it has been shown that the preferential homing of ILCs to the small intestine is controlled by diet-derived nutrients. ILCPs and mature ILC subsets express α4β7 integrin, CCR7, and CCR9 to varying degrees (85, 92) and, similar to T lymphocytes, ILC1, and ILC3 trafficking to the small and large intestine requires α4β7 integrin (85) that binds to MAdCAM-1, abundantly expressed on endothelial cells in the intestine. Furthermore, the Vitamin A metabolite retinoic acid is essential for the homing of ILC1s and ILC3s, but not ILC2s, to the small intestine (85). Specifically, it was found that ILC1s and ILC3s migrate from the bone marrow to mesenteric lymph nodes in a CCR7-dependent manner, where retinoic acid induces expression of α4β7 integrin and CCR9 (85), whose ligand CCL25 is abundant in the small intestine. In contrast, CCR9 expression is acquired by α4β7^+^ILC2Ps already in the bone marrow and therefore retinoic acid-independent (85, 100). This feature likely links the nutrient status of the host to the type of local immune response through the preferential migration of specific ILC subsets to the small intestine. This concept is consistent with the observation that lack of Vitamin A, as it occurs in malnutrition, causes a reduction of ILC3s and impaired protection against bacterial pathogens in the intestine, whereas ILC2s and anti-helminth responses are increased (16). This switch to type 2 barrier immunity likely ensures continued commensalism with evolutionary partners (helminths, commensal bacteria) in the small intestine during nutrient deficiency. In contrast, the homing of ILC3s to the colon requires α4β7 integrin, but not CCR9, and is therefore independent of retinoic acid (85). It has not been explored whether other chemotactic receptors used by T cells, such as GPR15 (108), enable ILC homing to the colon.

### Circulating ILCPs

ILCPs are present within tissues, such as the intestine and other organs, including blood, in mice and humans (9, 38, 40, 42, 109, 110). Despite the presence of ILCPs in both peripheral blood and tissues, parabiosis studies in mice indicate that ILCs other than NK cells in both lymphoid and non-lymphoid tissues are mainly tissue-resident (39). Subsequently, this concept has been challenged by the finding that human CD34^−^CD117^+^ ILCPs are present not only in blood, but also in a variety of lymphoid and non-lymphoid tissues (40), demonstrating that these ILCPs can leave the circulation and migrate into tissues. Furthermore, these circulating human CD117^+^ ILCPs can be considered the equivalent of naïve T cells since they lack immediate effector function, but have the ability to differentiate into mature ILC1s, ILC2s, and ILC3s *in vitro* and upon adoptive transfer into mice. ILCPs were therefore proposed to serve as cellular substrates for local “on-demand” ILC-poiesis within tissue (111). Further studies are needed to clarify potential species-specific differences in ILC migration/tissue residency between humans and mice and to establish to what extent mature ILCs in tissues are replenished by circulating ILCPs.

Progenitors upstream of human CD34^−^CD117^+^ ILCPs express the adhesion/homing receptor CD34 and are found in a variety of tissues, but not in blood (111). It is plausible that loss of CD34 expression on CD34^+^ ILCPs triggers the entry of CD34^−^CD117^+^ ILCPs into the circulation (111). Furthermore, IL-1β (in combination with IL-2 and IL-7) acts as a growth factor for CD117^+^ ILCPs *in vitro* (40) and it has been suggested that production of IL-1β induced by disruption of tissue homeostasis promotes the migration of ILCPs from blood into tissue (111). However, as a cytokine, IL-1β lacks direct chemotactic activity and therefore other, yet unknown, chemotactic guidance cues and their corresponding receptors must be involved.

### ILCP Egress From Bone Marrow

In mice, ILCPs and ILC2Ps, unlike common lymphoid progenitors, express the chemokine receptor CXCR6 and their egress from the bone marrow is partially dependent on CXCR6, thereby regulating ILCP entry into the circulation (92). In contrast, adult ILC3Ps migrate from the bone marrow to the periphery in a CXCR6-independent manner (9). Furthermore, a recent study demonstrated that IL-33 signaling is required for the egress of ILC2Ps from the bone marrow during the perinatal period by downregulating CXCR4 expression (37). Finally, the bioactive lipid sphingosine-1 phosphate (S1P) promotes lymphocyte egress from several organs (112) and S1P receptor 5 (S1PR5) is essential for the bone marrow egress of NK cells (97). However, it has not been investigated whether S1P receptors also regulate the egress of ILCPs from bone marrow.

### ILC Recirculation

Both mouse and human ILCPs in the blood express CD62L, which promotes lymph node entry of ILCPs, whereas lymph node exit requires S1P receptors (42). The later possibility is further supported by the finding that treatment with the S1P agonist FTY720, which disrupts S1P gradients and results in S1P receptor internalization from the cell surface (112), causes ILC-penia, while increasing the number of ILCs in lymph nodes (113). These studies are consistent with the concept that, similar to naïve T cells, ILCPs and some mature ILCs have the ability to re-circulate between blood and lymphoid organs (Figure 1). This notion is further supported by a recent study in mice, demonstrating that ILC1s (similar to NK cells) recirculate between blood and lymph nodes in a CD62L- and CCR7-dependent manner, whereas ILC3s in lymph nodes are mainly tissue-resident (43). Furthermore, among human ILCs, NKp44^−^ ILC3s, likely representing ILCPs (40), have higher expression of genes encoding surface receptors involved in lymphocyte recirculation (CD62L, CCR7, S1PR1) than NKp44^+^ ILC3s (114). Finally, compared to their circulating counterparts, human lymphoid tissue-resident ILC3s express CXCR5 and CCR7, known to regulate positioning within lymphoid organs (114).

### Tissue Retention of ILCs

Finally, less is known about the factors that retain ILCs within tissues once they are recruited. This likely involves the same receptors that are required for the tissue retention of T lymphocytes (115) (Table 1). For example, intestinal and skin ILCs express CD69 (41, 48, 92), which antagonizes the egress receptor S1PR1 (116). In addition, ILC2s from human tissues express the collagen-binding integrin CD49a (30) that has been shown to promote T cell retention in tissues. Populations of mouse ILC1s and ILC3s also express CD49a (86) as do human intraepithelial ILC1s in the intestine (50). In addition, the latter subset expresses CD103 (αE integrin) (50), which together with β7 integrin binds to E-cadherin on epithelial cells. CD103^+^ ILC2s have also been identified in mouse skin (117). Further studies (such as genetic ablation in mice) are required to demonstrate a direct role for specific receptors in the tissue retention of ILCs.

## ILC Positioning Within Tissue

**Figure 4:**
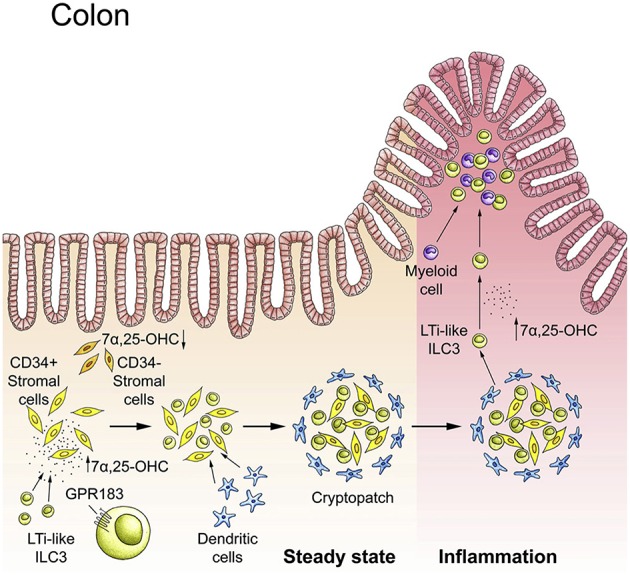
The GPR183-oxysterol pathway positions ILC3s in the colon. In steady-state, LTi-like ILC3s are recruited to cryptopatches by the receptor GPR183 that senses locally produced cholesterol metabolites (oxysterols). The GPR183 ligand 7α,25-dihydroxycholesterol (7α,25-OHC) is produced by CD34^+^ fibroblastic stromal cells. The interaction of GPR183^+^ LTi-like ILC3s with stromal cells leads to cryptopatch formation in steady-state. Inflammation increases 7α,25-OHC production and promotes the migration of GPR183^+^ ILC3s and myeloid cells to inflammatory foci within the colon. Adapted from reference (21).

ILCs occupy strategic positions within tissues to perform their organ-specific functions. Proper ILC positioning within tissue is also critical for the spatial compartmentalization of tissue immunity. For example, as discussed above, ILCs inhabit tissue-specific niches, which facilitates the interaction with other immune cells as well as with non-hematopoietic cells. However, there is very limited knowledge regarding the signals and receptors that direct ILCs to local tissue niches. Recent work elucidated how metabolic signals ensure that ILCs are properly positioned in the intestine to carry out their function. We found that intestinal ILC3s lacking the G protein-coupled receptor GPR183 (also known as EBI2) exhibit aberrant localization (21). GPR183 recognizes hydroxylated metabolites of cholesterol, so-called oxysterols, with 7α,25-dihydroxycholesterol as the main GPR183 ligand (118, 119). We demonstrated that oxysterols sensed through the receptor GPR183 function as guidance cues to position ILC3s within intestinal cryptopatches, which is critical for lymphoid tissue formation in the colon (21) (Figure 4). Similar findings have been subsequently reported by two other labs (95, 120). Chu et al. found that GPR183 also regulates ILC3 recruitment to the small intestine (but not to the colon), possibly through promoting α4β7 integrin surface expression on ILC3s (95). We further showed that oxysterols are produced by specialized stromal cells located within cryptopatches/isolated lymphoid follicles (21). The intriguing possibility that dietary cholesterol in breast milk is a source of oxysterols required for post-natal lymphoid organogenesis in the colon remains to be explored (119).

This complements previous work showing that CXCL16 produced by CX3CR1^+^ dendritic cells guides the positioning of CXCR6^+^ NKp46^+^ ILC3s to the villi of the small intestine, where they contribute to epithelial defense through the production of IL-22 (58). Notably, the chemokine receptors CCR6 and CXCR5 (and their respective ligands CCL20 and CXCL13), although specifically expressed by LTi-like ILC3s, are not required for ILC3 migration to cryptopatches, neither in the small nor large intestine (121, 122). However, it has recently been reported that skin ILC3s are positioned within hair follicles in a CCR6-dependent manner (87). In mesenteric lymph nodes, ILC3s are found in a specific anatomical location, the interfollicular region (84), and GPR183 also promotes the proper positioning of ILC3s to this region, whereas CCR6 and CXCR5 are dispensable (95, 96).

Apart from NK cells that largely lack GPR183, ILC subsets other than LTi-like ILC3s also express GPR183 to varying degrees (21). However, the specific functions of GPR183 in other ILC subsets is unknown. Overall, our recent work and that of others indicates an important role for local cholesterol metabolites in directing ILC migration.

Other lipids, such as leukotrienes and prostaglandins, are likely relevant for the intra-tissue localization of ILCs (Figure 3). For example, human ILC2s are phenotypically defined by the expression of CRTH2 (27), the prostaglandin D2 receptor, and prostaglandin D2 induces the chemotaxis of ILC2s *in vitro* (123). Accordingly, CRTH2 mediates accumulation of mouse ILC2s in the inflamed lung (98). Moreover, human ILC2s found in asthma are responsive to the lipid mediators prostaglandin D2 and lipoxin A4 (124). As mentioned above, amino acid derivatives, such as a tryptophan metabolites, can regulate ILC homeostasis through AHR. However, it is currently unknown whether amino acid-derived molecules can also act as chemotactic cues, guiding ILC positioning.

Apart from host-derived signals, the gut microbiota also likely regulates ILC migration and positioning through the local production of metabolites. For example, lymphoid-tissue resident commensal bacteria promote ILC3 localization to mesenteric lymph nodes and Peyer's Patches (125). Furthermore, the short-chain fatty acid butyrate controls the compartmentalization of ILC3s in Peyer's Patches (94). Specifically, it has been shown that butyrate, sensed through the receptor GPR109A on ILC3s, is more abundant in ileal than jejunal Peyer's Patches, thereby inhibiting the residence of CCR6^−^NKp46^+^ (and CCR6^+^NKp46^−^) ILC3s in ileal Peyer's Patches (94).

Overall, more metabolic signals remain to be identified that promote proper ILC localization in healthy and inflamed tissue. The use of lipid metabolites, rather than the exclusive use of genome-encoded proteins (chemokines), as guidance cues for ILCs within tissue might be advantageous for the host. Lipid metabolites can be rapidly produced and inactivated through enzymatic conversion, as exemplified by the GPR183 ligand 7α,25-dihydroxycholesterol since its synthesis from cholesterol is controlled by two enzymes, cholesterol 25-hydroxylase (CH25H) and 7α-hydroxylase (CYP7B1); and it can be further metabolized into bile acid precursors that lack chemotactic activity by the enzyme 3β-hydroxysteroid dehydrogenase type 7 (HSD3B7) (118). This allows tight regulation of 7α,25-dihydroxycholesterol abundance within tissue. Furthermore, lipid metabolites likely easily diffuse within tissue, thereby facilitating the generation of precise local chemotactic gradients. Finally, from the same precursor molecule, two bioactive metabolites with distinct functions can be generated: 25-hydroxycholesterol generated by CH25H from cholesterol has anti-viral and anti-inflammatory activity, whereas 7α,25-dihydroxycholesterol synthesized from 25-hydroxycholesterol by CYP7B1 regulates immune cell migration through GPR183 (118). This feature likely allows coordinated regulation of tissue-resident immune function by lipid metabolites.

## ILC Migration During Inflammation

During infection and other tissue insults, ILCs must migrate to local sites of inflammation within tissue. For example, Neuropilin-1^+^ human LTi cells are present in inducible bronchus-associated lymphoid tissue (iBALT) in the inflamed lung in chronic obstructive pulmonary disease (COPD) (99). A recent study showed that, in Mycobacterium tuberculosis infection, ILC3s are recruited via the CXCL13-CXCR5 axis to the lung, thereby mediating the formation of iBALT associated with granulomas, which contributes to early control of infection together with the production of IL-17 and IL-22 (91). In addition, IL-17-producing ILC3s are present in the alveolar space in asthma patients (126). Similarly, ILC2s are increased in the broncho-alveolar lavage fluid of humans with idiopathic pulmonary fibrosis (127). Furthermore, it has recently been shown that ILCs are recruited into the inflamed skin in a CCR6-dependent manner (43). Finally, the accumulation of LTi-like ILC3s in mesenteric lymph nodes after helminth infection is dependent on CCR7-mediated trafficking (84).

The GPR183-oxysterol pathway also plays an important role in controlling ILC migration in inflamed tissue (119). Mobilization of ILC3s from cryptopatches into the surrounding tissue occurs during intestinal inflammation (60). We showed that the recruitment of ILC3s (and myeloid cells) to inflammatory foci in the colon is dependent on GPR183 (21) (Figure 4). It is reasonable to assume that increased oxysterol synthesis induced by tissue injury conveys perturbation of tissue homeostasis to the immune system, initiating ILC movement and the inflammatory response (119). It is currently unknown whether other metabolites produced in inflamed tissue regulate ILC migration and localization.

An important feature of ILCs is their ability to contribute to the repair of tissues damaged by infection, inflammation, and irradiation, which is likely dependent on their local migration and accumulation within damaged tissues. For example, LTi-like ILC3s restore lymphoid tissue architecture after viral infection (128), promote thymic regeneration after irradiation (129), and protect the intestine from graft vs. host disease-induced damage after hematopoietic stem cell transplantation (55). Similarly, ILC2s alleviate virus-induced damage to the lung (33).

Moreover, a recent study established the new concept of ILC inter-organ trafficking during inflammation. Specifically, it was shown that inflammatory IL-25-responsive ILC2s can migrate from the intestine to the lung during helminth infection to support host defense (41). The exit of ILC2s from the intestine into the blood via the lymphatic system was mediated by S1P (41), the critical factor regulating lymphocyte egress from tissues (112). CD69, expressed on tissue-resident ILCs, antagonizes S1PR1 through downmodulation of S1PR1 from the cell surface (116). In contrast, inflammatory ILC2s are CD69^−^, allowing S1PR1-dependent egress into the circulation (41). A previous study in mice had found that intestinal NK cells, ILC1s, and ILC3s are CD69^hi^, whereas ILC2s are CD69^lo^, supporting the concept that intestinal ILC2s might be “less tissue-resident” than other intestinal ILC subsets (92). Finally, it has been suggested that signals from the local microbiota promote S1P receptor expression on ILC2s, thereby allowing them to exit the intestine (130).

It remains to be tested whether inter-organ trafficking of ILCs also occurs between other organs. In this context, it is relevant that in various inflammatory conditions activated ILCs are found in peripheral blood. For example, human NKp44^+^ ILC3s expressing homing receptors for skin and intestine appear in the circulation after conditioning for hematopoietic stem cell transplantation (131). Similarly, circulating ILC2s are increased in humans with asthma (132). These observations suggests that inter-organ trafficking might also occur in humans after mobilization of ILCs into the blood in response to inflammatory stimuli.

As outlined above, the recruitment of blood-borne ILCPs during infection may contribute to ILC heterogeneity within tissue. However, the signals activating blood-resident ILCs within tissue and recruiting them into the inflamed tissue from the circulation are unknown. This could involve tissue-derived signals sensed within the local vasculature and/or intra-tissue signals. Furthermore, the relative contribution of local expansion of resident ILCs vs. the recruitment of ILCPs to inflamed tissue is still unclear and may vary between tissues and the type of insult. For example, inflammatory signals could disrupt RANKL-RANK interactions, thereby allowing the local proliferation of CCR6^+^ ILC3s (61).

## ILC Migration in Cancer

Another largely unexplored area that warrants further investigation is ILC migration in cancer. The tumor microenvironment constitutes a unique metabolic milieu, resembling inflamed tissue. Among ILCs, NK cells are often the predominant population found in the tumor microenvironment, e.g., in human lung and colon cancer (30). Due to their cytotoxic activity, NK cells are a promising target for anti-cancer immunotherapy (133). However, in many human cancers, e.g., lung adenocarcinoma, NK cells are under-represented within the tumor compared to healthy tissue (134), especially the cytotoxic CD56^dim^ subset (135). This suggests that NK cell recruitment to tumors is suboptimal and targeting NK cell migration could be a valuable strategy in cancer immunotherapy. Several chemokines and their respective receptors mediating NK cell migration to tumors have been identified, such as CXCL8-CXCR1/CXCR2, CXCL10-CXCR3, CXCL12-CXCR4, and they are being explored as clinical targets (136).

Whereas, a role for NK cells in controlling cancer growth and metastasis has been well-established, the function of other ILC subsets in cancer, especially in regards to migration, is poorly understood (133). In several hematological malignancies, human ILC1s, ILC2s, and ILC3s are increased in the blood compared to healthy individuals (133), supporting the notion that ILCs can be mobilized into the circulation during malignancy. There is also some evidence that circulating human ILC2s could contribute to immunosuppression in gastric cancer (137).

Furthermore, ILC3s with likely LTi function are enriched in solid tumors both in mice and humans. For example, NKp46^+^ ILC3s invade B16 mouse melanoma expressing IL-12 (138). It was shown in the same model that lymphoid tissue-resident (splenic) Rorc^fate−map+^ ILCs have superior anti-tumor activity than intestinal or hepatic Rorc^fate−map+^ ILCs and Rorc^fate−map−^ ILC1s/NK cells (139). NKp46^+^CCR6^+^CXCR5^+^ ILC3s with LTi properties are also enriched in tumor-associated tertiary lymphoid structures in human non-small cell lung cancer (140). Both studies showed that tumor-associated ILC3s upregulate adhesion molecules on the tumor vasculature, which likely promotes anti-tumor immunity through the recruitment of T cells. These tertiary lymphoid structures are of interest because of their importance for T cell-mediated anti-tumor immunity and their general positive prognostic value for cancer outcome, e.g., in lung cancer (141). However, tumor-associated lymphoid structures may also promote cancer growth. For example, one study reported that high amounts of CCL21 recruit CD4^+^ LTi cells to tumors in a CCR7-dependent manner, which is associated with the formation of tumor-promoting lymphoid-like stroma in melanoma (89). In a mouse model of breast cancer, it was also shown that CCL21 recruits CCR7-expressing ILC3s with an LTi phenotype (CD4^+^CCR6^+^) to the tumor environment (90). Furthermore, CXCL13 was required for the clustering of CXCR5^+^ ILC3s with mesenchymal stromal cells in the tumor microenvironment, which supported lymph node metastasis (90).

Interestingly, complementary to our findings in the intestine, it has recently been shown that oxysterol recognition through GPR183 is required for the development of iBALT (142), a common feature of active lung inflammation. However, this study did not determine the role of GPR183-expressing ILCs in this process. Overall, it seems plausible that the oxysterol-GPR183 pathway could also be involved in the formation of tertiary lymphoid structures in cancer.

## Concluding Remarks

ILCs maintain healthy organ function and it is increasingly recognized that ILC function is critically dependent on their trafficking to and localization within tissues. Accordingly, ILC migration and the mechanisms of ILC tissue recruitment are areas that are beginning to be explored in more depth. It is important to comprehensively identify the guidance cues and receptors that control ILC localization and motility in tissues. In the long-term, cell surface receptors regulating ILC migration to inflamed or malignant tissues could serve as new therapeutic targets for human diseases.

## Author Contributions

The author confirms being the sole contributor of this work and has approved it for publication.

### Conflict of Interest Statement

The author declares that the research was conducted in the absence of any commercial or financial relationships that could be construed as a potential conflict of interest.
